# Therapeutic Potential of Phytoconstituents in Management of Alzheimer's Disease

**DOI:** 10.1155/2021/5578574

**Published:** 2021-06-08

**Authors:** Anurag Kumar Singh, Sachchida Nand Rai, Anand Maurya, Gaurav Mishra, Rajendra Awasthi, Anshul Shakya, Dinesh Kumar Chellappan, Kamal Dua, Emanuel Vamanu, Sushil Kumar Chaudhary, M. P. Singh

**Affiliations:** ^1^Centre of Experimental Medicine & Surgery, Institute of Medical Sciences, Banaras Hindu University, Varanasi 221005, Uttar Pradesh, India; ^2^Centre of Biotechnology, University of Allahabad, Prayagraj 211002, India; ^3^Department of Medicinal Chemistry, Institute of Medical Sciences, Banaras Hindu University, Varanasi 221005, Uttar Pradesh, India; ^4^Amity Institute of Pharmacy, Amity University Uttar Pradesh, Noida 201303, Uttar Pradesh, India; ^5^Department of Pharmaceutical Sciences, Faculty of Science and Engineering, Dibrugarh University, Assam 786004, Dibrugarh, India; ^6^Department of Life Sciences, School of Pharmacy, International Medical University (IMU), Bukit Jalil, Kuala Lumpur 57000, Malaysia; ^7^Discipline of Pharmacy, Graduate School of Health, University of Technology Sydney (UTS), Ultimo, New South Wales, Australia; ^8^Faculty of Biotechnology, University of Agronomic Science and Veterinary Medicine, 59 Marasti Blvd, 1 District, 011464, Bucharest, Romania; ^9^Faculty of Pharmacy, DIT University, Mussoorie-Diversion Road, Makkawala, Dehradun 248 009, Uttarakhand, India

## Abstract

Since primitive times, herbs have been extensively used in conventional remedies for boosting cognitive impairment and age-associated memory loss. It is mentioned that medicinal plants have a variety of dynamic components, and they have become a prominent choice for synthetic medications for the care of cognitive and associated disorders. Herbal remedies have played a major role in the progression of medicine, and many advanced drugs have already been developed. Many studies have endorsed practicing herbal remedies with phytoconstituents, for healing Alzheimer's disease (AD). All the information in this article was collated from selected research papers from online scientific databases, such as PubMed, Web of Science, and Scopus. The aim of this article is to convey the potential of herbal remedies for the prospect management of Alzheimer's and related diseases. Herbal remedies may be useful in the discovery and advancement of drugs, thus extending new leads for neurodegenerative diseases such as AD. Nanocarriers play a significant role in delivering herbal medicaments to a specific target. Therefore, many drugs have been described for the management of age-linked complaints such as dementia, AD, and the like. Several phytochemicals are capable of managing AD, but their therapeutic claims are restricted due to their lower solubility and metabolism. These limitations of natural therapeutics can be overcome by using a targeted nanocarrier system. This article will provide the primitive remedies as well as the development of herbal remedies for AD management.

## 1. Introduction

### 1.1. Features and Epidemiology of Alzheimer's Disease

Alzheimer's disease (AD) is a seriously worrisome disease for the well-being of people who have it and for their caregivers—throughout the world—with a critical socioeconomic liability. It is a familiar type of dementia. It represents around 60%–80% of different types of dementia [1]. Victims of AD commonly exhibit irreversible degeneration and progressive loss in the neurons of the entorhinal cortex and hippocampus along with several degrees of cognitive functions, namely, trouble in recollecting names, ongoing occasions, and comorbid behavioral dysfunctions, like depression. In advanced phases, motor nerve degeneration occurs, like trouble in talking, gulping, and strolling, which can inevitably prompt the passing away of the person [1,2]. It has been assessed that worldwide there are 35 million individuals suffering from AD and dementia and by 2030 the figure may rise to around 65 million, and it might get multiplied by 2050 [1, 3]. Various factors, such as decreased physical action, infection, smoking, and occurrence of diseases like obesity and diabetes, are a threat to the emergence of AD [4]. The various potential mechanisms are blood-brain barrier (BBB) disruption [5], impaired brain metabolism [5], cellular autophagy impairment [6], unsettling influence of calcium homeostasis and expanded oxidative stress [7], expanded neuroinflammation [8], and neuronal apoptosis [9], at least one of described pathologies could be present, causing AD. Generally, patients suffer from late-onset AD, an irregular type of AD; however, less than 1% of the prevailing cases are characterized as early-onset AD, a genetic type of AD. Genetically linked AD is associated with the mutated amyloid precursor protein (APP) and/or presenilin-1 (PS-1) and presenilin-2 (PS-2) genes. The mutation in the PS genes is brought about by mixing natural and hereditary variables and acknowledging polymorphism in the “*ε*4 allele” of the apolipoprotein E (APOE) gene [10].

### 1.2. Pathophysiology of Alzheimer's Disease

Major pathological signs of AD include amyloid-containing senile plaques amassing in the extracellular site, activation and accumulation of the microtubule-associated protein tau- (MAPT-) driven neurofibrillary tangles (NFTs) inside the cell, and proximity of persistent neuronal inflammation in the infected parts. The various assumptions, together with the cholinergic, amyloid, and tau assumptions (Table 1), identify with the etiology of the disease [7, 25]. Amyloid hypothesis is considered the most significant theory in which irregular preparation of *β*-amyloid (A*β*) and/or injury of its systemic clearance are accountable for AD growth-associated phenotypes. A*β* is produced by the action of proteolytic enzymes, that is, *β*- and *γ*-secretases on the APP, although A*β* overproduction and/or potential impedance of its dispensation may bring about its extracellular aggregation. Indeed, surplus production of A*β* is a regular feature of a genetically linked AD or early-onset AD, whereas the late-onset AD is recognized by the deficiency in A*β* clearance as opposed to its excess production [26]. In any case, such disorganization in A*β* homeostasis triggers the formation of the A*β*-senile plaques, which in turn irreversibly stimulate the astrocytes and microglia. Chronic alteration in glial cell functionality intervenes with the structural and functional integrity of the associated neurons, leading to neurodegeneration and impairment of the fate of neurotransmitters, which ultimately results in the development of clinical indications of AD [27]. Additionally, NFTs are an example of the major features of pathological AD. The building block proteins of the cytoskeletal and the signal transduction pathways of neurons amassed with NFTs are hampered drastically due to impairments in the protein posttranslational changes, such as phosphorylation of structural microtubule proteins and functional tubulin-associated proteins. Furthermore, MAPT, a significant component of NFTs, is seen to be hyperstimulated in the brains of AD patients [28]. Several kinases are involved in the phosphorylation of the tau protein. Besides, the serine-threonine protein kinases, namely, c-Jun N-terminal kinase (JNK), cyclin-subordinate kinase 5 (CDK5), glycogen synthase kinase 3*β* (GSK3*β*), and p38, are proline-directed, which triggers the stimulation of the Thr-Pro or Ser-Pro tau motifs, by phosphorylating them [29], thus prompting missorting of the tau to the somatodendritic location, which disturbs the microtubule dynamics, along with tau polymerization and accumulation in its hyperphosphorylated structure [28]. The fibrils and tau oligomers feature as the initiators of the inflammatory response in the microglia [30], and the thickness of NFTs corresponds to the retardation in cognitive abilities in a person suffering from AD [10]. Even though A*β* is greatly considered as a pathological sign of AD pathophysiology, numerous ongoing proposals are involved in the dismissal of this hypothesis. However, a range of preclinical as well as clinical interventions have failed to prove the significance of targeting A*β* as a therapeutic modality [2, 31], and, hence, more research is being done on tau pathology and neuroinflammation [25], as previously studies have rarely done any research on the link between A*β* and tau in AD. The ongoing pattern has included another keyword called “neuroinflammation”; this approach is expected to have enormous scope in the etiology of AD, unequivocally upheld by the genetic, clinical, and preclinical examinations [4, 32]. Central nervous system (CNS) inflammation is recognized as neuroinflammation, which is activated by immune cells of the brain (like astrocytes and microglia) as well as periphery-derived immune cells (like white blood cells), trailed by the discharge of secondary messengers, because of certain damage nearby or as a reaction to inflammatory components [33, 34]. Inflammatory procedures in the brain are generally strongly controlled when compared with the peripheral tissues [35]. In any case, risk factors of AD, like diabetes, obesity, infections, smoking, and decreased physical action, may offer a thrust to systemic inflammation, leading to disruption of the BBB followed by the progressive inflammatory response in the resident immune cells of the brain [4, 36]. Although microglia, the macrophages of the brain, are the main immune effector cells in CNS, other kinds of cell-like oligodendrocytes, astrocytes, endothelial cells, and nonmicroglial myeloid cells may take an interest in the inflammatory procedures [4, 34]. Many outcomes speculate that impairment in the immune response may be a causative aspect in AD idiopathy [37] as well as a reason for neuron loss and may subsequently effect cognition and neuronal plasticity [34, 38, 39]. At the molecular level, astrocytes and microglia activate the inflammatory response cycle by activating the immunoregulatory mechanisms like proinflammatory cytokines and the complement system [40, 41]. Microglia has a scope for biological functions, for example, synaptic plasticity maintenance, synaptogenesis, CNS development, antigen presentation, pathogen recognition, and phagocytosis [42]. These proinflammatory cytokines and associated complement systems have a progressive and destructive impact on the brain of AD patients. Besides, microglia keeps control to eliminate A*β* deposits. These inflammatory mediators furthermore trigger/stimulate the impairment in the cognition level as well as the necrotic impact on the neuron, that is, cytotoxicity [42]. Furthermore, two types of stimulation patterns have been exhibited by these cells: classical activation (M1 phenotype) and alternative activation (M2 phenotype). Classical activation stimulation is portrayed by discharging TNF-*α* and interleukins (IL), although another stimulation arbitrates the anti-inflammatory effects, which favors AD [39, 42].

### 1.3. Current Therapeutic Approaches for Alzheimer's Disease Patients

There was no innovative medication endorsement for AD later in 2003 [31], and the present treatment choices provide only symptomatic relief [32, 33]. Acetylcholinesterase inhibitors such as galantamine, donepezil, and rivastigmine are utilized to refine memory and consideration in AD patients assisting in expanding the intensities of acetylcholine by forestalling its break at the synapsis. While galantamine and rivastigmine are utilized for slight-to-modest AD, donepezil is utilized for all phases of AD. Tacrine (TAC) is the major prescription given for AD treatment, as an AChE inhibitor. As it was receding from the market due to its hepatic toxicity at prescribed dosages, it has shown an extensive margin as the most utilized AChE inhibitor in the progress of multitarget anti-AD drugs. Another alternative for modest-to-severe AD which is appropriate is recognized as the N-methyl-D-aspartate (NMDA) receptor antagonist, with slight side effects, for example, dizziness, gastrointestinal irritation, and headache [32]. During various potential therapeutics, including numerous disease-modifying agents, these came into the clinical trials, and many of them are in phase III. Importantly, Aducanumab, ANAVEX2-73, ALZT-OP1a/b, CAD106, Crenezumab, and E2609 cover a fair proportion of these developing drugs, that is, 61% of the total drugs under phase-III trial. A majority of these therapeutic candidates aim at amyloid-related pathologies. Be that as it may, different targets, for example, neurotransmitter-based, tau-based, antioxidant-based, and anti-inflammation-based targets, are additionally under clinical trial. Two of these preliminaries are focusing on inflammatory pathways and recommend neuroinflammation as a significant causative element for AD-related pathophysiology [31].

## 2. Medicinal Plants against Alzheimer's Disease

A detailed description of various classes of plants utilized in AD therapy is given below (Table 2).

### 2.1. Ashwagandha (*Withania somnifera* (L.) Dunal (Solanaceae))

Ashwagandha belongs to the family of Solanaceae and generally its root is utilized for medicinal purposes. It is ordered as a *Rasayana* (rejuvenating) and is accepted to have cell reinforcement movement, free radical rummaging action, and a capacity to bolster a solid resistant framework [74, 75]. The alkaloid-rich fraction of the *Withania* root showed soothing action on the CNS of many mammals, specifying its role to create relaxation. The withanamides consisted of glucose and serotonin, and the long-chain hydroxyl fatty acid moieties have been seen to scavenge unobstructed radicals produced through the commencement and advancement of AD. It was also observed that withanamides obstructed the activated neuronal cell death by amyloid plaques [73, 76–78]. The Ashwagandha aqueous extract was found to improve the cholinergic action, which helped to stimulate the acetylcholine amount and choline acetyltransferase activity in mice, which might be the perception of enhancing memory activity [79, 80]. Ashwagandha methanol extract was found to trigger the growth and development of the neuronal network in human neuroblastoma cells [79, 81]. Sehgal et al. described that *per-oral* intake of *Withania* root extract overturned behavioral shortages and A*β* plaque formation in validated AD mice. Such effects of Ashwagandha were associated with the upregulation of the low-density lipoprotein receptor-linked protein in the liver [82]. The roots of Ashwagandha advance the defense mechanism of the body in case of chronic disease, by boosting cellular immunity along with neutralizing the lethal mediators of the cytotoxic cascade, namely, cytokines and reactive oxygen species (ROS) [83, 84].

### 2.2. Brahmi (*Bacopa monnieri* (L.) Wettst. (Scrophulariaceae))

**Figure 1:**
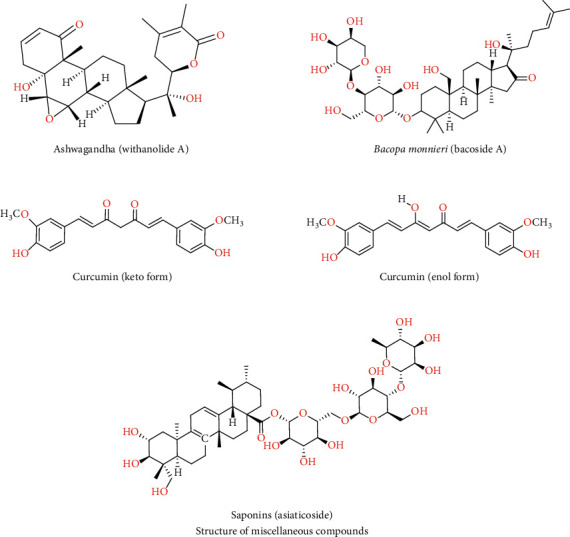
Structure of some active compounds against AD.

It is an unpleasant tasting creeper plant, generally utilized in *Ayurveda* as a nerve tonic, for insomnia and so many ailments [85, 86]. *Brahmi* (family: Scrophulariaceae) contains several branches with reduced oblong leaves and purple flowers. Its main constituents are saponins and triterpenoid bacosaponins (Figure 1). *Bacopa monnieri* (BM) acts by lowering the activity of divalent metals, sifting ROS, reducing lipid peroxides formation, and obstructing lipoxygenase movement [87]. It is traditionally employed for regulating memory and cognitive function and improving neuropharmacological and nootropic activities [88–91]. They secure cortical, hippocampal, and striatal neurons from the DNA-linked dysfunctions and associated neurotoxicity in AD. *Bacopa monnieri* has been also reported to decrease cholinergic deterioration and cognition-improving actions in rats experimentally induced with AD-like features [92].

### 2.3. Turmeric (*Curcuma longa* L. (Zingiberaceae))

*Curcuma longa* (family*:* Zingiberaceae) improves detoxification of the liver, regulates cholesterol level, excites the metabolic process, and improves immunity. Turmeric is used as an alimentary spice, where symptoms of AD are reported, 4.4-fold less than other regions in Southeast Asian countries [93]. The active component of turmeric is its oil and water-soluble curcuminoids comprising curcumin [94]. Curcumin is the primary curcuminoid, containing 3-methoxy-4-hydroxy substituents and two feruloyl moieties. Its terminal side units are joined by an unsaturated seven-carbon bond that attaches a *β*-diketo function, which results in a symmetric curcumin molecule. Curcumin occurs in two probable tautomeric forms, enol and diketo, based on the pH (Figure 1) [95, 96].

### 2.4. Shankhpushpi (*Convolvulus prostratus* Forssk. (Convolvulaceae))

Shankhpushpi is employed as a nervine stimulant for upgrading memory and cerebral function in different formulae in India [85, 97, 98]. Shankhpushpi metabolites provide nootropic and memory stimulating action, together with improving the pharmacological function [99]. Ethanol extract of CP and its fractions (ethyl acetate and aqueous) notably enhance learning and memory in rats [100]. Many studies show that administration of CP extracts enhances the memory in aged mice and improves the retention and spatial learning performance, demonstrating memory-upgrading in neonatal rat pups.

### 2.5. Gotu Kola (*Centella asiatica* (L.) Urban (Apiaceae))

Gotu Kola, commonly named Brahmi, belongs to the family Apiaceae. The principal constituents of Gotu Kola are saponins (asiaticosides). It is commonly used to purify the blood, enhance memory, reduce blood pressure, and promote life. Gotu Kola helps calm the mind and dismiss tension. In *Ayurveda*, Gotu Kola aqueous extracts are applied for rejuvenating and restoring neural cells and to improve insomnia [101]. By-products from Gotu Kola (asiatic acid and asiaticoside) are potent antioxidant compounds and prevent ROS-induced neuronal death. Furthermore, *in vitro* findings suggest that Gotu Kola is efficient in hindering the *β*-amyloid cell; thus, it is effective in the management of poison from *β*-amyloid in AD patients [102].

### 2.6. Guggulu (*Commiphora*)

Guggulu is an oleogum resin oozing from splits, gaps, or cuts from the bark of many plant species, such as *Commiphora mukul* (Hook. ex Stocks) Engl. (Burseraceae), *Commiphora myrrha* (T.Nees) Engl. (Burseraceae), *Commiphora kua* (R.Br. ex Royle) Vollesen (Burseraceae), and *Commiphora wightii* (Arn.) Bhandari (Burseraceae). It is light yellow or earthy colored having a sweet-smelling scent and harsh astringent taste. It consists of a blend of 30% to 65% water-soluble gum, 25% to 40% alcohol-soluble resins, and approximately 8% volatile oils, water-soluble components, plus mucilage, protein, and sugar. Alcohol-soluble excipients are commiphoric acids, heerabomyrrhols, and commiphorinic acid. It also contains phenols, ferulic acid, and nonphenolic aromatic acids, which are effective antioxidants, and they are valuable for AD [103–105].

### 2.7. Jyotishmati (*Celastrus paniculatus* Willd. (Celastraceae))

Jyotishmati is a significant plant valued for its quality, which can be improved for a long time. It is employed for enhancing memory and maintaining cerebral activities [106]. *Celastrus paniculatus* (CP) extricates secured neuronal cells against H_**2**_O_**2**_^−^-actuated poison to some extent because of their cancer prevention activity and capacity to activate antioxidant proteins. *Celastrus paniculatus* also extracts secured neuronal cells from glutamate-induced toxicity, by controlling the glutamate receptor activities. The cholinergic activity of CP seed aqueous extract was observed in a dose-dependent manner thereafter, refining memory activity [107].

### 2.8. Jatamans*i* (*Nardostachys jatamansi* (D. Don) DC. (Caprifoliaceae))

Jatamansi is a very rich plant that is used in medicine, and it is respected in *Ayurveda*. The principal part of the *Nardostachys jatamansi* (NJ) is the rhizome or root, which has therapeutic value. It contains different types of coumarins and sesquiterpenes. NJ extract showed a decrease in the signs of chronic fatigue syndrome in rats. The alcoholic extract of *Nardostachys jatamansi* showed significant improvement in the learning and memory of both young and aged mice [108].

### 2.9. Ginkgo (*Ginkgo biloba* L. (Ginkgoaceae))

The leaf extract of *Ginkgo biloba* is an extensively used medicinal plant, which belongs to the Ginkgoaceae family. The primary phytoconstituents of ginkgo are comprised of terpenoids and flavonol glycosides. Its leaf extract is commonly used for age-related memory disorders in several European and Asian countries [109]. *G. biloba* extract is extensively employed for treating patients with numerous forms of dementia in Europe [110, 111]. *G. biloba* nanosized extract improves acetylcholine neurotransmitter discharge from several sections of the brain compared to control group animals. Its nanosized extract shows enhanced bioavailability and a superior immersion level [112]. The preclinical experiment concludes that ginkgo reduces the oxygen radical release and proinflammatory purpose of macrophages and decreases the corticosteroid creation and proliferation, glucose uptake and utilization, and adenosine triphosphate (ATP) production [113]. Ginkgo also seems to increase the flow of blood, increasing red blood cell deformability and reducing red cell accumulation, encouraging nitric oxide production, and alienating platelet-activating factor receptors [113].

## 3. Phytoconstituents against Alzheimer's Disease

**Figure 2:**
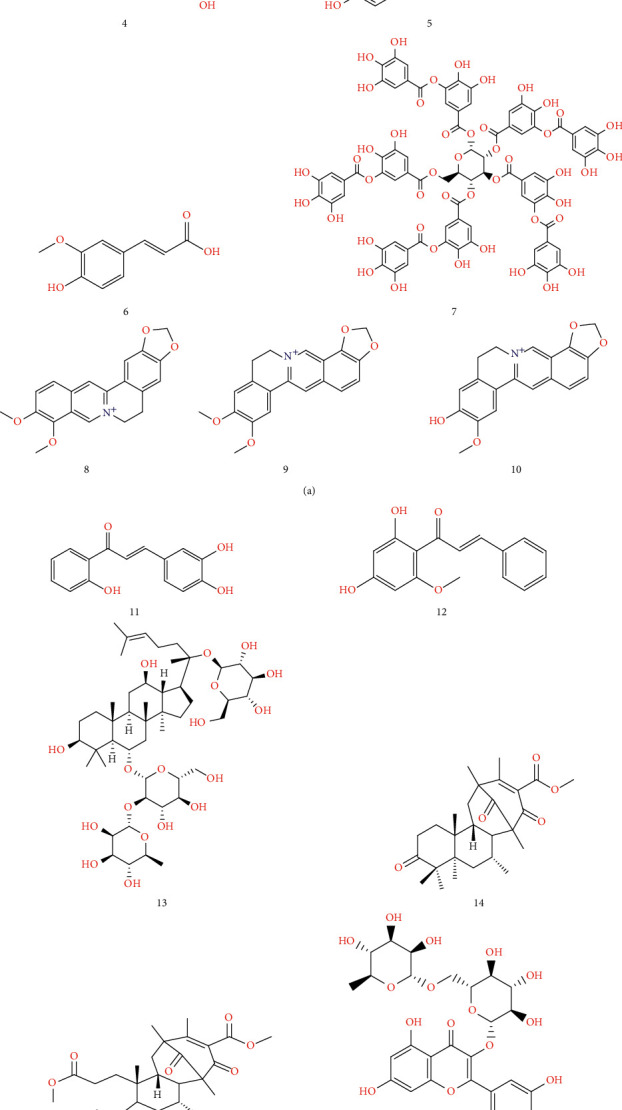
Phytoconstituents against AD

A detailed description of various phytoconstituents reported for the cure of AD is given below (Figure 2).

**Baicalein (1)** is a potent antioxidant obtained from *Scutellaria baicalensis* Georgi (Lamiaceae), which is employed in Traditional Chinese Medicine (TMC). Baicalein shows more powerful anti-BACE1 activity [IC_50_ = *µ*M] when compared with quercetin and luteolin [114]. Baicalein has been also reported to cause A*β*-oligomerization and fibrillation and inhibition of A*β*-induced toxicity in PC12 cells and disaggregation of preformed A*β* amyloid fibrils [115]. Baicalein oral intake decreases the BACE1 protein level [116] and also its continual use improves A*β* deposition in both the N2a-Swedish APP cells and the neuronal cell group. Moreover, it exhibits BACE1-reducing activity (IC_50_ of 50 *µ*M) [11, 117]. Quercetin has been found to reduce BACE1 expression in medicated male C57BL/6 strain mice and, thus, negatively controls the amyloidogenic treating of *β*APP [118].

**Myricetin (3)** polyphenolic compound, also referred to as hydroxyquercetin, shows neuroprotective effects against neuronal cell injury tempted by A*β* [119]. The hydrophobic nature and low molecular weight of myricetin enhance the crossing of BBB, which provides an excellent therapeutic environment [120]. Shimmyo et al. [119] have pointed out that myricetin has a dual role, which reduces BACE1 activity (IC_50_ = 2.8 *µ*M), exclusive of affecting protein expression, and activates the *α*-secretase (ADAM10) in the cell-free enzyme activity. The outcome of myricetin on neuronal cells has been found to be lower than anticipated.

**Genistein (4)** reduced the BACE1 action in a dose-dependent manner (IC_50_ = 6.3 *µ*M). Genistein also reduced A*β*-induced inflammation and cell death in *in vivo* and cell-based studies [121–123]. Another study indicated that a lower concentration of genistein crossed the BBB without affecting neurotoxicity [124].

**Salvianolic acid (Sal B) (5)** is a derivative of caffeic acid, obtained from the *Salvia miltiorrhiza* Bunge (Lamiaceae) root, found to disaggregate preformed fibrils in a minimized A*β* fibril formation. Sal B also modulates BACE1 activity in SH-SY5Y-APPsw and decreases A*β* production in H4-SwedAPP, N2a-SwedAPP, and HEK-BACE1 cells [125–127].

**Ferulic acid (6)** obtained from *Oryza sativa* L. (Poaceae), *Triticum aestivum* L. (Poaceae), and other fruits and vegetables has been seen to reduce the A*β* groups at a concentration of 1.57 *µ*M in *in vivo* and *in vitro* studies [128]. Ferulic acid also reduces the BACE1 enzymatic action and points to BACE1 stability, affecting mRNA expression level [129].

**Oral intake of tannic acid (7)** at 30 mg/kg/day dosage in transgenic PSAPP mice enhances the diminishing behavior, decreases cerebral amyloidosis, and enhances antiamyloidogenic *β*APP processing without exhibiting any side effects [13].

**Berberine (8)** is an isoquinoline alkaloid, isolated from *Coptis chinensis* Franch. (Ranunculaceae), having neuropharmacological properties, which regulate *β*APP processing, resulting in a decline of the A*β* protein. Berberine is administered orally at a dosage of 25 or 100 mg/kg per day in transgenic AD mice, for four months, which indicates considerable mitigation of A*β* pathology and lack of influencing BACE1 protein levels [130].

**Epiberberine and Groenlandicine (9 and 10)** are protoberberine alkaloids that inhibit BACE1 activity with IC_50_ values of 8.55 and 19.68 *µ*M, respectively [131].

**2,2,4-Trihydroxychalcone acid (TDC) (11)**, a derivative of Chalcone-flavanoids, isolated from *Glycyrrhiza glabra* L. (Fabaceae), reduces BACE1 activity (Ki value = 3.08 *µ*M; IC_50_ = 2.5 *µ*M). TDC reduces production of the A*β*40 and A*β*42 stages in the HEK293-APPswe cells by efficiently overcoming the BACE1 activity on the *β*APP. TDC does not exhibit any impact on *α*- and *γ*-secretases, effectively impedes A*β* in cells by restraining BACE1 activity, and does not affect any BACE1 protein levels in cell-based assays and *in vivo* (B6C3-Tg mice) studies [132].

**Cardamonin (12)** isolated from the *Boesenbergia rotunda* (L.) Mansf. (Zingiberaceae) shows potent inhibition (IC_50_ = 4.35 ± 0.38 *µ*M) and does not alter the TACE (*α*-secretase). *In silico* studies with −9.5 kcal/mol results indicates its affinity to strongly bind with enzymes and it has been verified to cross the BBB easily [133]. Oral intake of cardamonin for 30 weeks at a dose of 10 mg/kg does not show any superficial toxicity, thus acknowledging its safe consumption [134].

**Ginsenosides (13)**, a phytoconstituent of *Panax ginseng* C.A.Mey. (Araliaceae), show a decrease in BACE1 activity. Ginsenosides are reported to decrease BACE1 activity and BACE1 expression, without any action on the total APP and sAPP*α* levels in *in vitro* testing [135].

**Asperterpenes A and B (14 and 15)** meroterpenoids are obtained from *Aspergillus terreus* Thom. and soil-derived mold. They show strong BACE1 inhibitory actions in HEK-BACE1 cells. The IC_50_ values of asperterpenes A and B are found to be 78 and 59 nM, respectively. Asperterpene A (70 nM) lowers A*β*42 development and prevents BACE1 action in the HEK-293 and N2a-APP cell lines [136].

**Rutin and Galangin (16 and 17)** are extracted from *Fagopyrum esculentum* Moench (Polygonaceae) and *Alpinia officinarum* Hance (Zingiberaceae) and, hence, act as *β*APP-selective BACE1 inhibitors and show a capacity to unstabilize BACE1 division [137].

**Andrographolide (18)** is a labdane diterpenoid extracted from *Andrographis paniculata* (Burm. F.) Nees (Acanthaceae). It shows the inhibiting property against glycogen synthase kinase-3*β* (GSK-3*β*) as well as reduction of the phosphorylated tau protein and amyloid-beta aggregate maturation in aged degus [138–140].

## 4. Nanocarrier Containing Natural Product Used to Combat Alzheimer's Disease

**Figure 3:**
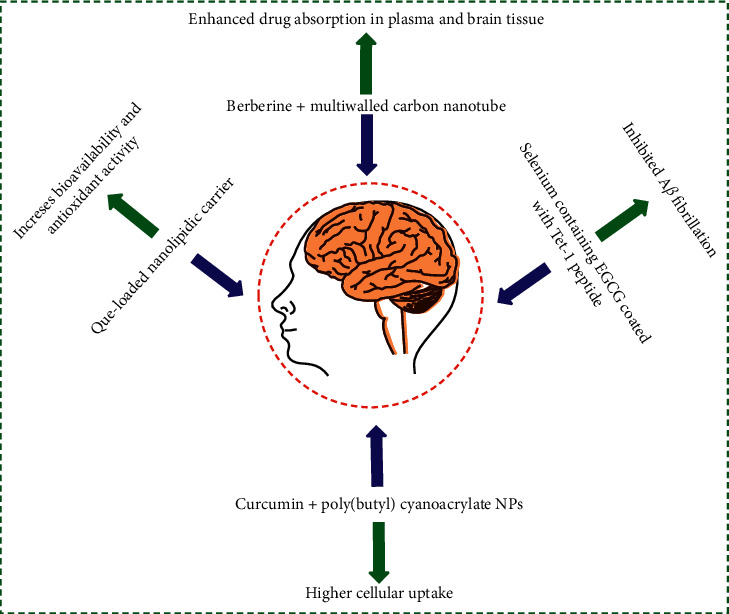
Delivery of herbal nanoformulation and its effects.

In today's scientific community, the main challenge is to deliver a drug to its specific site. Nanocarriers play a very important role in delivering the medicaments to any target specifically (Figure 3). Several drugs have been reported for the management of age-linked complaints such as dementia, AD, and the like. Toxicity and safety are the major limitations associated with the use of synthetic drugs; thus scientists are focusing on the practice of natural bioactive AD management. Many phytochemicals are capable of managing AD, but their therapeutic claims are restricted due to their lower solubility and metabolism, caused by their large molecular size. These limitations of natural therapeutics can be overcome by using a targeted nanocarrier system.

### 4.1. Curcumin

Curcumin is a polyphenol compound reported for its neuroprotective property, having good action against neurological disorders. Mulik et al. developed apolipoprotein E3-mediated poly(butyl) cyanoacrylate NPs containing curcumin, which showed higher cellular uptake and phosphatability by its sustained release behavior [141]. Tiwari et al. developed a nanoparticle of curcumin-loaded poly(lactic-co-glycolic acid) (PLGA), which showed activity toward the induction of neurogenesis, by internalization into the hippocampus neuronal stem cell [142]. They also enhanced the gene expression involved in cell proliferation and neuronal differentiation in comparison to pure curcumin. This study also revealed that curcumin nanoparticles increased neuronal differentiation by activating the Wnt/*β*-catenin pathway, involved in the regulation of neurogenesis. Furthermore, Mathew et al. prepared curcumin-loaded PLGA nanoparticles and coupled them with a Tet-1 peptide, which had a retrograde transportation ability, so that it easily penetrated the BBB and the prepared formulation that destroyed the *β*-amyloid aggregates and also showed its antioxidative property with a nontoxic effect [143]. Encapsulation of curcumin within the PLGA showed the indestructible inherent property of curcumin. Cheng et al. prepared nanocurcumin with PLGA-loaded micelles of average size of 80 nm, which showed improved bioavailability in the brain of Tg2576 mice in comparison to pure curcumin. It also showed a good effect in the treatment of AD_s_. They have almost 100 percent entrapment efficiency, with 37.6% loading of curcumin [144]. Malvajerd et al. did a relative study of a nanolipid carrier (NLC) and solid lipid nanoparticle (SLN), and the results revealed a high brain uptake and bioavailability of NLC with C_max_ of 390.3 ng/g, AUC of 505.76 ng/g h, and *T*_max_ of 1 h of the curcumin [145]. Mourtas et al. prepared a lipid-polyethylene glycol polylactide PEG-curcumin derivative, which reduced A*β* aggregation and enhanced penetration of curcumin in a postmortem patient sample [146]. Zhao et al. developed curcumin-conjugated carboxybetaine methacrylate nanoparticles, which were found to be effective against A*β*_42_ fibrils in comparison to pure curcumin [147]. Huo et al. developed the selenium nanosphere and curcumin selenium-PLGA nanosphere and they found that the result was in favor of the curcumin selenium-PLGA nanosphere, as compared to the selenium nanosphere in the AD mice model [148]. Kakkar and Kaur 2011 prepared curcumin-loaded solid lipid nanoparticles and they reported a neuroprotective effect against the aluminum-induced mice brain, with alteration in behavioral, histopathological, and biochemical parameters [149]. Hoppe et al. prepared a curcumin-loaded lipid core nanocapsule, which was observed to produce reduction in the hyperphosphorylation of tau and A*β* in AD_s_, when compared with pure curcumin [150]. Meng et al. prepared a curcumin-loaded low-density lipoprotein mimic nanostructure, with lactoferrin. It was administered to AD_s_ animals, and a drastic change was noticed in AD_s_ progression, with increased concentration in the brain and higher bioavailability [151].

### 4.2. Epigallocatechin-Gallate

Many treatments show symptomatic relief with numerous side effects in AD_s_, but none can halt their development. So, for targeting a broader target, not only the symptom but also the pathology with less side effects, a green tea polyphenol-derived active constituent epigallocatechin-gallate **(**EGCG), has given a lot of hope and acted as a potential agent for therapeutic application. Singh et al. (2018) prepared an EGCG nanoparticle and studied the neurobehavior and pathophysiology of the aluminum chloride-induced rat model [152]. The results showed that neurobehavioral impairment was significantly increased and neuritic plaque and neurofibrillary tangles were absent, and a lower level of biochemical immunohistochemical protein was found, which was the major cause for the induction of AD_s_. Cano et al. developed a nanoparticle-containing EGCG and ascorbic acid and found enhanced stability when the EGCG and ascorbic acid nanoparticle was administered orally to the mice model, and also the resultant EGCG accumulation in the organ, including the brain, was higher. It was found to be five times more concentrated than free EGCG in the long term, from 5 to 25 hours [153]. The known model APPswe/PS1dE9 (APP/PS1) mice were used in the study and it was also observed that there was a remarkable increase in synapses and decrease in the burden of amyloid *β* plaque. Hoyos-Ceballos et al. prepared EGCG loaded PLGA/PF127 nanoparticle, nearly 100 nm in size, with an encapsulation efficiency of 86%. The finding on the oxidative stress model showed rotenone-induced ROS generation prevention and mitochondrial membrane potential loss, with DNA fragmentation in the nerve cells [154]. Hence, all observations supported that EGCG had great therapeutic efficacy toward AD_s_. Smith et al. prepared EGCG-containing lipid nanoparticle that was delivered to the mouse model, and they observed good ability to promote the amyloid precursor protein by *α*-secretase upregulation, which resulted in inhibition of *β*-amyloid plaque formation [155]. The bioavailability of nano-EGCG was found to be two times greater than free EGCG, and neuronal *α*-secretase increment was up to 91%. Zhang et al. prepared the selenium-containing EGCG covered by a Tet-1 peptide; it decreased EGCG cytotoxicity and inhibited A*β* fibrillation, with disaggregation of the A*β* fibrils into nontoxic compounds [156].

### 4.3. Berberine

Berberine is an isoquinoline alkaloid that shows a neuroprotective effect and it is used for the management of different age-related neurological disorders like dementia, AD_s_, mental depression, schizophrenia, and anxiety. The major mechanism of berberine to treat AD_s_ is inhibition of acetylcholinesterase and indoleamine 2,3-dioxygenase. In 2017, Lohan and coworkers prepared berberine containing multiwalled carbon nanotubes, having a surface coating of polysorbate and phospholipid [157]. The size of the prepared formulation was 186 nm with an absorption capacity of 68.6%. They obtained a significant improvement of drug absorption in plasma and brain tissue of the rat *vis-à-vis* a pure drug. Enhanced recovery was observed in the memory performance of rats on days 18–20. A good reduction in the *β*-amyloid plaque was noticed. Soudi et al. synthesized surface-modified berberine chitosan nanoparticles modified with the tween 80, PEG4000, and miltefosine against lipopolysaccharide [158]. The synthesized formulation showed the neuroprotective behavior against lipopolysaccharide-activated cerebral and associated liver changes in rats.

### 4.4. Quercetin

It is a flavonoid, having a neuroprotective effect with potency toward scavenging ROS, as well as some drawbacks, like lesser solubility and lower bioavailability. Kumar et al. prepared a Que-loaded nanolipid carrier delivered to the brain, which enhanced bioavailability and antioxidant activity [159]. Quercetin- (Que-) solid lipid nanoparticles were delivered for the aluminum-induced neurotoxicity, and a significant betterment in memory retention was observed in animal models of AD_s_ [160]. Kuo et al. prepared ApoE-Que-RA-PA liposome, with intended transport via the BBB, for recovery of neurotoxicity of AD_S_ model. This formulation also minimized acetylcholinesterase activity and A*β* plaque formation *in vivo* [161]. Phachonpai et al. developed Que liposomes for nasal delivery. The study reported lowering of cholinergic neurons by a drop in oxidative stress in the AD model [162]. Aluani et al. studied the nanoencapsulated Que, which showed neuroprotective behavior when compared with free Que, in the neuronal model of oxidative stress injury [163].

### 4.5. Resveratrol

It is a polyphenolic agent from the stilbene compound, which has low bioavailability and poor solubility in water and is very fast. Frozza et al. reported a Resveratrol-loaded lipid nanocarrier, which modulated *in vitro* initiation of A*β* inflammation [164]. In another study in 2013, Frozza et al. prepared Resveratrol LNC, which showed destruction in A*β*_1–42_ in a rat model [165]. Sun et al. formulated Resveratrol-loaded mesoporous nanoselenium, which affected memory impairment and also inhibited A*β* aggregates and oxidative stress [166]. Similarly, Lu et al. established the polymeric micelles loaded with Resveratrol, which inhibited A*β*-induced damage of cells by oxidative stress [167].

### 4.6. Piperine

It is an alkaloid obtained from piper species fruits which especially acts on the central nervous system and is specifically evident on acetylcholine. Low bioavailability, high lipophilic potential, and poor aqueous solubility make Piperine different from others. Elnaggar et al. prepared monoolein cubosome-loaded Piperine, modified it with tween, and administered it to the AD_s_ model, which showed remarkable efficacy and restored cognitive function in comparison to the free drug [168]. Etman et al. prepared microemulsion containing Piperine and achieved high therapeutic efficacy, with higher brain delivery in AD_s_ model, compared to free Piperine [169]. Yusuf et al. prepared Piperine-loaded SLN_s_ coated with PS80, which decreased the SOD level, with enhanced acetylcholinesterase and reduced plaques and tangles, while studying the histopathology [170]. There are multiple nanocarriers used to treat Alzheimer's disease. These nanocarriers incorporate active medicament, either herbal or synthetic, which act on different hypotheses causing Alzheimer's, such as, tau protein, *β*-amyloid, and oxidative stress.

### 4.7. Polymeric Micelles

They are specially designed to prevent the hydrophobic nature of the AD drug. Kulkarni et al. established a brain-targeting nanopolymeric structure employing polymeric n-butyl-2-cyanoacrylate (PBCA) and encapsulating the radio-labeled amyloid affinity drug I-clioquinol [171]. The loaded NPs excellently crossed the BBB in a mouse model and improved the brain retention of I-CQ-PBCA NPs. Chitosan-covered PLGA nanoparticles (NPs) conjugated with a novel anti-A*β* antibody were prepared by Jaruszewski et al., which displayed boosted BBB uptake and better targeting of the A*β* proteins *in vitro*. Due to its enhanced spreadability and stability, there is a possibility to develop a nanocarrier for analysis and cure of AD [172].

### 4.8. Liposomes

These are developed to enhance the bioavailability of drugs, increase their solubility, lower their toxicity, and so forth. Mourtas et al. premeditated diverse liposomes, incorporating a derivative of curcumin and anti-transferrin antibody (TrF) as a BBB transport mediator. Liposomes containing curcumin derivatives and/or anti-TrF displayed elevated affinities for amyloid deposits in AD patient's brain samples [146].

### 4.9. Cubosomes

They are constricted with biodegradable carriers with liquid crystalline structures. They contain a three-dimensional structure with two aqueous phases, within which bioactive ingredients are incorporated. Elnaggar et al. prepared tween-integrated monoolein cubosomes (T-cubs) enveloped with Piperine and studied their capabilities on the AD brain. Preclinical investigation in rats proved its effectiveness in improving cognitive skills and delaying the progression of AD [168].

### 4.10. Lipid Nanoparticles

They are designed to combat the drawbacks of the other developed nanocarrier systems. This type of particle shows a good release pattern with stability. Bernardi et al. reported the effectiveness of indomethacin-loaded lipid-core nanocapsules (IndOH-LNCs) to prevent the neuroinflammatory and neurotoxic effect of A*β*1-42 on the neuronal cells, possibly via the enhanced release of endogenous anti-inflammatory cytokines, that is, interleukin-10 and condensed glial activation and c-Jun N-terminal kinase phosphorylation [173].

## 5. Conclusion

Herbal remedies have long been used for the management of AD in the form of extracts, phytoconstituents, and their nanocarriers. These have appeared as capable candidates for the cure of AD and other related disorders. It might be proved that botanicals are a prosperous resource of a newer class of bioactive materials for the controlling of neurodegenerative illnesses. The therapeutic efficacy of *Withania, Bacopa, Curcuma, and Mucuna pruriens* (L.) DC. (Fabaceae) is not only limited to AD but also beneficial for the treatment of Parkinson's disease (PD). *Withania, Bacopa, Curcuma, and Mucuna pruriens* exhibit potent therapeutic potential in PD [174–179]. Furthermore, the *in vivo* activity is yet to be revealed in animals as well as human models to validate their curative effectiveness in AD and other associated ailments. It is expected that herbal remedies have the scope for developing some novel compounds in the drug development process for AD management. This current article focuses on the types of neurological hypotheses for AD and the role of herbal remedies to manage it.

## Figures and Tables

**Table 1:** Neurological hypothesis and effect of phytoconstituents in AD.

Types of hypothesis	Class	Natural compounds	Pharmacological/mechanism of action	References
Secretase hypothesis	Neuronal cells and cell-free system	Myricetin	Reduced the generation of A*β*	[11]
			It also decreased the production of A*β* via upregulation of the *α*-secretase actions and/or downregulation of *β*-secretase actions	[12]
		Tannic acid	Prevention of cognitive decline, inhibition of the activity of *β*-secretase, and reduction of the AD-like pathology in transgenic mice	[13]
A*β* aggregation	Biflavonoids	Taiwaniaflavone and monoflavonoid apigenin	Observed that the biflavonoid was more effective at decreasing the extension of A*β* fibril	[14]
Nontoxic A*β* oligomers	Oligomers	Small molecules of flavonoids	Soluble A*β* oligomers are the main toxic species of A*β* triggering neurodegeneration; A*β* oligomers over monomeric fibrillary A*β* are started as active therapeutics for nullifying and pointing A*β* toxicity in case of AD	[15–17]
A*β*-induced neurotoxicity	Polyphenols and flavonoids	Quercetin	Inhibiting the formation of A*β* fibril, quercetin was less effective in terms of improving the toxicity of A*β* as compared to myricetin	[18]
Tau hyperphosphorylation	Glycogen synthase kinase 3 beta (GSK-3*β*)	Linarin	Prevents the neurotoxicity induced by A*β* (25–35) via PI3K/Akt activation, which can subsequently lead to promotion of Bcl-2 regulation and inhibition of GSK-3*β*. It plays a key role in neuroprotection and AChE inhibition	[19]
Oxidative stress	Biflavonoid	Morelloflavone	Potential to act as a potent inhibitor of lipid peroxidation	[20]
	Natural flavonoid	*Silybum marianum*	Promotes the viability of neuron upon hydrogen peroxide insult	[21, 22]
RAGE	Polyphenols	*Camellia sinensis*	RAGE plays a role as inducer of oxidative stress (OS) and AD pathophysiology	[23, 24]

**Table 2:** Medicinal plants against AD.

Drug/family	Phytoconstituents	Mechanism of action	Application	Enzymatic assay/target organism/cell line	References
*Ginkgo biloba*/Ginkgoaceae	Terpene trilactones, ginkgolides A, B, C, J and bilobalide, polyprenols biflavones, proanthocyanidins, alkylphenols	Cell damage in Alzheimer's, decrease in fluid behaviour of membrane	Treating dementia with 240 mg daily, applied for boosting memory	C57BL/6 mice by inhibiting LPS-induced rises in iNOS levels	[43–45]
*Salvia officinalis*/Lamiaceae	Cineole, borneol, fumaric acid, chlorogenic acid thujone, tannic acid, oleic acid, ursolic acid, cornsole, caffeic acid, nicotinamide	Inhibition of acetylcholinesterase	Treating mild-to-moderate Alzheimer's	……..	[46–48]
*Rosmarinus officinalis*/Lamiaceae	Carnosic acid, rosmarinic acid, camphor, caffeic acid, ursolic acid, betulinic acid, rosmaridiphenol	Inhibitor of lipid peroxidation	Protection of brain from stroke helpful to avoid Alzheimer's	…….	[49–51]
*Curcuma longa*/Zingiberaceae	Curcumin and polyphenol	Involves inhibition of articular NF-B, a transcription factor activated in vascular endothelium	Alzheimer's disease treatment inhibition of acetylcholinesterase and tau protein	Transgenic APPSw mouse model (Tg2576)	[52–54]
*Zingiber officinale*/Zingiberaceae	Zingerone, shogaols, gingerols, *β*-sesquiphellandrene, bisabolene, farnesene, citral, cineol	Inhibit the synthesis of prostaglandin-E2 (PGE2) and thromboxane B2, act on serotonin receptor	Treatment of Alzheimer's	SHSY-5Y cells from A*β*-42 toxicity	[55–57]
*Urtica dioica*/Urticaceae	Acetylcholine, histamine, 5-hydroxy tryptamine, protein, fat, fiber	Boosting up cholinergic system in the brain	Treating Alzheimer's disease	…..	[58–60]
*Lepidium meyenii*/Brassicaceae	Alkaloids, amino acids, arginine, histidine, phenylalanine, threonine, tyrosine, anthocyanins, glucotropaeolin 81	It provides its antioxidant and AChE inhibitory activities 82	Enhance learning and memory, decrease lipid peroxidation and acetylcholinesterase	Ovariectomized mice	[61–63]
*Huperzia serrata*/Huperziaceae	Lycoposerramine-H, serratidine, obscurumine A, 11*α*-O-acetyllycopodine, huperzine A, huperzine B, huperzinine, lycodine	Acetylcholinesterase (AChE), inhibitory activity	It is used in neurodegeneration, Alzheimer's disease	Activates MAPK/ERK signaling pathway in mice	[64–66]
*Terminalia chebula*/Combretaceae	Arjunglucoside I, arjungenin, chebuloside I and II, chebulinic acid, gallic acid, ethyl gallate, punicalagin	Acetylcholinesterase inhibition	Used possibly in treatment of Alzheimer's	……..	[67–69]
*Withania somnifera*/Solanaceae	Withanolides, dehydrowithanolide R, withasomniferin A, withasomidienone, withasomniferols A to C, withaferin A, and withanone	Neuronal cell death started by amyloid plaques is blocked	Alzheimer's disease	Rat neuronal cells (PC-12)	[70–72]
*Bacopa monnieri*/ Scrophulariaceae	Bacoside A, bacoside, betulinic acid, D mannitol, stigmastanol, b sitosterol, stigmasterol	Showed cognition-enhancing effect in a rat model of Alzheimer's and also inhibited cholinergic degeneration inhibition	Boosting memory and treating Alzheimer's is potential use	Rat model of AD	[73]

## Abbreviations

3*β* or GSK: 3*β* glycogen synthase kinase 3*β*
AChE: Acetylcholinesterase
AD: Alzheimer's disease
APOE: Apolipoprotein E
APP: Amyloid precursor protein
ATP: Adenosine triphosphate
A*β*: *β*-Amyloid
BM: *Bacopa monnieri*
CDK5: Cyclin-subordinate kinase 5
CNS: Central nervous system
CP: *Convolvulus pluricaulis*
CT: *Clitoria ternatea*
DNA: Deoxyribonucleic acid
EGCG: Epigallocatechin-gallate
EOAD: Early-onset AD
IL: Interleukins
IndOH-LNCs: Indomethacin-loaded lipid-core nanocapsules
JNK: Jun N-terminal kinase
LOAD: Late-onset AD
MAPT: Microtubule-associated protein tau
NFTs: Neurofibrillary tangles
NJ: *Nardostachys jatamansi*
NLC: Nanolipid carrier
NMDA: N-Methyl-D-aspartate
NPs: Nanoparticles
PEG: Polyethylene glycol
PS-1: Presenilin-1
PS-2: Presenilin-2
ROS: Reactive oxygen species
SLN: Solid lipid nanoparticle
TAC: Tacrine
TCM: Traditional Chinese Medicine
TrF: Anti-transferrin antibody.
